# Evaluation of an *in vitro* fibre fermentation method using feline faecal inocula: repeatability and reproducibility

**DOI:** 10.1017/jns.2017.22

**Published:** 2017-05-24

**Authors:** Guido Bosch, Lisa Heesen, Karine de Melo Santos, Wilbert F. Pellikaan, John W. Cone, Wouter H. Hendriks

**Affiliations:** 1 Animal Nutrition Group, Wageningen University, PO Box 338, 6700 AH Wageningen, The Netherlands; 2 Faculty of Veterinary Medicine, Utrecht University, PO Box 80·151, 3508 TD Utrecht, The Netherlands

**Keywords:** Cats, Fibre fermentability, *In vitro* methodology, Gas production, SCFA, CP, citrus pectin, FOS, fructo-oligosaccharide, GG, guar gum, *R*_max_, maximum rate of gas production, SBP, molassed sugar beet pulp, WM, wheat middlings

## Abstract

To gain knowledge on the precision of an *in vitro* method for characterisation of the fermentability of dietary fibres, this study aimed to evaluate the repeatability and reproducibility of such a method. Substrates used were citrus pectin (CP), fructo-oligosaccharides (FOS), guar gum (GG), sugar beet pulp (SBP) and wheat middlings (WM). Each substrate was incubated with faecal inoculum from five cats with three replicates for each substrate–cat combination. Gas production was measured continuously during the 48 h incubation and SCFA and organic matter disappearance (only SBP and WM) were determined after incubation. Four consecutive runs were performed. The within-run variability (repeatability) was generally lower for the more simple and pure substrates (CP, FOS, GG) than for the more complex substrates containing mixtures of fibres (SBP, WM). Replicates showed high variability, in particular for SCFA profiles and parameters of gas production kinetics. The between-run CV (reproducibility) for the measured parameters were, in general, below 10 % for CP, FOS and GG and higher values were obtained for SBP and WM. It is concluded that for precise dietary fibre characterisation, the number of replicates should be multiple and adjusted according to the variability of the parameters of interest and the complexity of fibres. The method yielded reproducible results with some variation in absolute values obtained, which may have an impact on the significance level of the differences among substrates.

Dietary fibres may affect cats’ health, digestive processes and faecal characteristics^(^^1^^)^. Such effects depend on the properties of dietary fibres including their potential fermentability by the intestinal microbiota. The fermentability of dietary fibres can be characterised by *in vitro* methods that simulate intestinal fermentation. The methodology includes incubation of the fibrous substrate of interest with a faecal inoculum from the target animal species. Various considerations for such *in vitro* methods have been addressed^(^^2^^)^ including the required number of faecal donors, which is described in a companion article for cats using similar laboratory procedures and substrates as in the present study^(^^3^^)^. As with any analytical method, it is important that the results obtained from an *in vitro* fermentation method are repeatable and reproducible. The repeatability is defined as the measure of variation for analyses within the same run, whereas reproducibility is the variation among runs^(^^4^^)^. Although these types of precision are routinely evaluated for standard laboratory analyses, few studies have specifically examined these for *in vitro* fermentation methods. This study aimed to evaluate the repeatability and reproducibility of an *in vitro* method for characterisation of the fermentability of dietary fibres for cats.

## Experimental methods

### Substrates

Dietary fibres or fibre sources were selected based on their use in cat foods, contrasting chemical composition and anticipated fermentation characteristics^(^^5^^–^^7^^)^. Substrates were citrus pectin (CP; rapidly and highly fermentable, HM Rapid, TIC Gums), fructo-oligosaccharide (FOS; rapidly and highly fermentable; Orafti® IPS, BENEO-Orafti), guar gum (GG; rapidly and highly fermentable, 8/22, TIC Gums), molassed sugar beet pulp (SBP; slowly and highly fermentable; Research Diet Services) and wheat middlings (WM; slowly and moderately fermentable; Research Diet Services).

### Animals, housing and care

A total of five neutered female European shorthair cats (3 to 5 years old), with a mean body weight of 3·4 (sd 0·4) kg were used during this 4-week study. Cats did not receive any antibiotics for at least 6 months prior to faecal collections. The cats were part of a larger colony where cats are housed in group rooms with inside and outside areas. For the detailed design of the group rooms and the climate and light schedules, see Van Rooijen *et al.*^(^^8^^)^. Cats were fed a nutritionally complete (i.e. meeting the FEDIAF standards) commercial dry extruded diet (Perfect Fit In-Home; Mars Petcare) for at least 4 weeks prior to the first faeces collection. Each cat was fed individually in its own metabolic cage^(^^8^^)^ between 08.30 and 09.30 hours (about 45 % of their daily portion), 12.00 and 13.30 hours (about 10 %) and at 16.30 hours (about 45 %). The amount of food provided was appropriate to maintain optimal body weight. In the morning and afternoon cats went to their group room and in the evening and night cats stayed in their own cage. Water was always available *ad libitum*. Litter trays were only present in the metabolic cage and contained non-absorbent polyethylene litter (Katkor^®^; Rein Vet Products). The tray and litter were sterilised with 70 % ethanol on the day of faeces collection. The health status of the animals was monitored daily and cats were weighed weekly. The Animal Care and Use Committee of Wageningen University, Wageningen, the Netherlands Animal approved all care and experimental procedures.

### Preparation of inoculum and incubation

Faeces were transferred within 15 min of defecation to sterile 250 ml plastic bottles prefilled with CO_2_ and 250 ml of CO_2_ was immediately added. The bottle with faeces was closed and transported within 5 min to the analytical laboratory where faeces were processed to inoculum under a constant stream of CO_2_. Faeces from cats were not pooled but processed for each cat. Attached litter particles were manually removed from faeces and faeces were diluted 1:9 (w/v) in a 39°C anaerobic sterile physiological saline solution (9 g/l NaCl). The diluted mixture was homogenised for 60 s using a hand-blender and filtered through nylon fabric (pore size 40 µm, permeability 30 %; PA 40/30, Nybolt). The filtrate was mixed with a pre-warmed (39°C) N-containing medium^(^^9^^)^ in a 5:84 mixture (v/v) and gently flushed for 5 min with CO_2_. The resulting medium/inoculum mixture was dispensed (89 ml) into pre-warmed and CO_2_-flushed 250 ml serum bottles (Schott) containing 0·5 g of substrate. Bottles were then placed in the water-bath (39°C) and attached to a fully automated gas production equipment^(^^10^^)^, which recorded gas production for 48 h. This incubation time is longer than the average total tract transit times observed in cats (young adult cats 36 (sd 14) h; senior cats 26 (sd 6) h)^(^^11^^)^, with a orocaecal transit time of approximately 5 h^(^^12^^)^. However, 48 h was estimated to be required for characterising the fermentation kinetics of SBP and WM. After 48 h of incubation, fermentation liquids were sampled for determination of SCFA (i.e. acetate, propionate, butyrate, iso-butyrate, valerate, iso-valerate) concentrations and for organic matter disappearance (only for SBP and WM). All incubations were done in triplicate. In addition, the study was repeated resulting in four runs performed over 4 weeks (one run/week).

### Chemical analyses

All substrates were chemically characterised as described by Bosch *et al.*^(^^3^^)^. SCFA analyses as well as organic matter disappearance were analysed as described by Bosch *et al.*^(^^13^^)^.

### Calculations and data analyses

Gas (ml) and SCFA (mmol) productions were expressed per g organic matter (OM). Monophasic models^(^^14^^)^ were fitted to the data for cumulative gas production and the maximum rate of gas production (*R*_max_ in ml/(g OM h)) and the time at which it occurred (*T*_max_ in h) were calculated^(^^15^^)^. Acetate, propionate and butyrate were expressed as percentage of total SCFA as was the branched-chain proportion (iso-butyrate + iso-valerate). Mean and standard deviation values were calculated for all replicates of each substrate–cat combination within the four runs. CV_r_ (standard deviation/mean × 100) were computed to describe the repeatability of the method. For the evaluation of the reproducibility, the values of replicates for each substrate–cat combination within a run were averaged and then the average for each substrate within that run was computed. The between-run mean and standard deviation values were calculated and used to compute the CV_R_ (standard deviation/mean × 100). For production of SCFA and gas, differences among substrates within each run were tested for significance by ANOVA with Tukey pairwise comparisons.

## Results and discussion

All cats remained healthy throughout the study except for one cat that produced watery diarrhoea in the week of run 2, which was not used in the study. The substrates contrasted in terms of fermentation parameters measured, as was anticipated (for parameter values, see Bosch *et al.*^(^^3^^)^). Of the 285 SCFA analyses performed, six yielded false results. Fitting of the model for gas production was not possible for SBP and WM.

The total SCFA showed highly repeatable results for the more simple and pure substrate (CP, FOS, GG) whereas SBP showed a slightly higher median CV_r_ value, and CV_r_ value for WM was higher than 10 % (Table 1). Also the maximum CV_r_ values were higher for the more complex substrates (SBP, WM). Acetate and propionate proportion results were highly repeatable for all substrates, but for the butyrate proportion considerably higher median CV_r_ values were obtained. The values for branched-chain proportion were intermediate. Considerably high maximum CV_r_ values for the proportions of SCFA were found. Outliers in SCFA profiles were not excluded in the present study, as deviating values may represent normal biological variation. For example, the proportions of acetate, propionate and butyrate for CP in the four runs were, on average, 69·1, 20·7 and 5·4 %, but in five out of fifty-six fermentation liquids the proportions were 51·1, 29·5 and 9·0 %. Such values were obtained in one of three replicates and were not associated with a specific cat or run. Furthermore, incubation with FOS, GG, SBP and WM resulted also in consistent alternative profiles. These observations may relate to selective growth of specific microbial communities with one of two distinct metabolic behaviours. This hypothesis is in line with the bimodal distribution of specific taxonomic groups found within the human faecal microbiota^(^^16^^)^. For precise analysis of SCFA profile of a substrate, it is important to have at least three replicates, although additional study is required to explore these observations in more detail and determine the minimal number of replicates for precise characterisation of dietary fibre fermentability.

**Table 1.** Coefficients of variation (%) within four runs of *in vitro* fermentation parameters for fibrous substrates using feline faecal inocula*

		Substrate				
Parameter		CP	FOS	GG	SBP	WM
Total SCFA	Median	3·0	2·4	2·6	5·3	14·2
	Maximum	5·4	9·2	7·9	13·8	35·6
Acetate proportion	Median	1·5	3·6	3·5	2·7	5·8
	Maximum	19·7	26·7	24·5	26·1	30·0
Propionate proportion	Median	3·8	5·7	3·2	4·2	6·0
	Maximum	24·0	17·7	12·6	31·9	12·7
Butyrate proportion	Median	9·8	7·7	14·3	13·5	21·8
	Maximum	48·6	43·7	48·4	42·6	79·5
BCP	Median	6·0	7·4	5·7	7·4	11·8
	Maximum	59·6	48·2	46·9	55·1	52·6
Gas	Median	4·4	4·4	5·4	5·7	10·2
	Maximum	11·7	14·6	13·3	15·0	24·1
*R*_max_	Median	19·4	16·4	21·0	–	–
	Maximum	45·2	51·2	54·0	–	–
*T*_max_	Median	11·6	7·8	23·3	–	–
	Maximum	79·7	26·8	85·0	–	–
OMD	Median	–	–	–	2·7	5·0
	Maximum	–	–	–	26·6	15·4

CP, citrus pectin; FOS, fructo-oligosaccharide; GG, guar gum; SBP, molassed sugar beet pulp; WM, wheat middlings; BCP, branched-chain proportion of total SCFA production; *R*_max_, maximum rate of gas production; *T*_max_, time at which *R*_max_ occurred; OMD, organic matter disappearance.

* Indicated CV values (%) are based on nineteen sets of three replicates per substrate–cat combination in four incubation runs.

The CV_r_ values for gas production and organic matter disappearance showed a similar pattern as total SCFA produced and parameters of gas production kinetics showed considerably higher values. These values were higher than those obtained by Van Laar *et al.*^(^^4^^)^. In the latter study, CV_r_ values were based on duplicate evaluations of four substrates (soyabean meal, wheat grain, grass silage, maize gluten meal) in three to five runs by five laboratories with rumen fluid as the inoculum source. For gas produced after 72 h, CV_r_ values ranged between 1·4 and 10·3 % and for the parameters of gas production kinetics, similar CV_r_ values were obtained.

The CV_R_ values were for the measured parameters were below 10 % for the simple and pure substrates (CP, FOS, GG) and higher values were obtained for SBP and WM (Table 2). The organic matter disappearance values for SBP and WM were highly reproducible. The relatively high CV_R_ values for *R*_max_ were in line with those reported for parameters of fermentation kinetics by Van Laar *et al.*^(^^4^^)^. The CV_R_ values for SCFA and gas production were in line with those reported by McBurney & Thompson^(^^17^^)^ who incubated four substrates (oat bran, wheat bran, red kidney bean and GG) with faeces from one healthy human volunteer for 24 h on three separate non-defined occasions. The CV_R_ ranged from 3 to 10 % for SCFA production whereas that for gas production ranged from 1 to 16 %. It should be noted that the reproducibility values in the present study relate to a relatively short time period applied between runs and might deviate when longer intervals are applied.

**Table 2.** Coefficients of variation (%) between four runs of *in vitro* fermentation parameters for fibrous substrates using feline faecal inocula*

	Substrate				
Parameter	CP	FOS	GG	SBP	WM
Total SCFA	4·4	2·4	5·0	7·8	8·3
Acetate proportion	3·8	4·7	7·0	7·6	11·8
Propionate proportion	9·6	6·2	9·5	11·9	14·5
Butyrate proportion	4·9	7·7	8·4	12·5	6·8
BCP	12·0	9·2	11·4	21·8	19·3
Gas	3·3	4·3	3·6	10·4	13·3
*R*_max_	10·5	5·9	10·7	–	–
*T*_max_	4·1	4·0	6·8	–	–
OMD	–	–	–	5·9	1·2

CP, citrus pectin; FOS, fructo-oligosaccharide; GG, guar gum; SBP, molassed sugar beet pulp; WM, wheat middlings; BCP, branched-chain proportion of total SCFA production; *R*_max_, maximum rate of gas production; *T*_max_, time at which *R*_max_ occurred; OMD, organic matter disappearance.

* Indicated CV values (%) are based on four incubation runs.

Of the ten substrate comparisons within each run, consistent patterns among the four runs were found. For total SCFA, four comparisons were significant (*P* < 0·05) and two non-significant. For gas production this was, respectively, six and one, acetate proportion five and one, propionate proportion six and one, butyrate proportion zero and seven, and branched-chain proportion four and three. Fermentation parameters for WM were generally different from the other substrates whereas the significance of the differences between the other substrates was more inconsistent.

In conclusion, for precise dietary fibre characterisation, number of replicates should be further explored and adjusted according to the variability of the parameters of interest and the complexity of fibres. The method yielded reproducible results with some variation in obtained absolute values, which may have an impact on the significance level of the differences among substrates.
